# Acquired nintedanib resistance in FGFR1-driven small cell lung cancer: role of endothelin-A receptor-activated ABCB1 expression

**DOI:** 10.18632/oncotarget.10324

**Published:** 2016-06-29

**Authors:** Bernhard Englinger, Daniela Lötsch, Christine Pirker, Thomas Mohr, Sushilla van Schoonhoven, Bernd Boidol, Charles-Hugues Lardeau, Melanie Spitzwieser, Pál Szabó, Petra Heffeter, Irene Lang, Margit Cichna-Markl, Bettina Grasl-Kraupp, Brigitte Marian, Michael Grusch, Stefan Kubicek, Gergely Szakács, Walter Berger

**Affiliations:** ^1^ Institute of Cancer Research, Department of Medicine I, Medical University of Vienna, Austria; ^2^ CeMM Research Center for Molecular Medicine of The Austrian Academy of Sciences, Vienna, Austria; ^3^ Department of Analytical Chemistry, University of Vienna, Vienna, Austria; ^4^ Institute of Organic Chemistry, Research Centre for Natural Sciences, Hungarian Academy of Sciences, Budapest, Hungary; ^5^ Division of Cardiology, Department of Medicine II, Medical University of Vienna, Vienna, Austria; ^6^ Institute of Enzymology, Research Centre for Natural Sciences, Hungarian Academy of Sciences, Budapest, Hungary

**Keywords:** small cell lung cancer, FGFR1, nintedanib, ABCB1, endothelin-A receptor

## Abstract

Genomically amplified fibroblast growth factor receptor 1 (FGFR1) is an oncogenic driver in defined lung cancer subgroups and predicts sensibility against FGFR1 inhibitors in this patient cohort. The FGFR inhibitor nintedanib has recently been approved for treatment of lung adenocarcinoma and is currently evaluated for small cell lung cancer (SCLC). However, tumor recurrence due to development of nintedanib resistance might occur. Hence, we aimed at characterizing the molecular mechanisms underlying acquired nintedanib resistance in FGFR1-driven lung cancer. Chronic nintedanib exposure of the FGFR1-driven SCLC cell line DMS114 (DMS114/NIN) but not of two NSCLC cell lines induced massive overexpression of the multidrug-resistance transporter ABCB1. Indeed, we proved nintedanib to be both substrate and modulator of ABCB1-mediated efflux. Importantly, the oncogenic FGFR1 signaling axis remained active in DMS114/NIN cells while bioinformatic analyses suggested hyperactivation of the endothelin-A receptor (ET_A_R) signaling axis. Indeed, ET_A_R inhibition resensitized DMS114/NIN cells against nintedanib by downregulation of ABCB1 expression. PKC and downstream NFκB were identified as major downstream players in ET_A_R-mediated ABCB1 hyperactivation. Summarizing, ABCB1 needs to be considered as a factor underlying nintedanib resistance. Combination approaches with ET_A_R antagonists or switching to non-ABCB1 substrate FGFR inhibitors represent innovative strategies to manage nintedanib resistance in lung cancer.

## INTRODUCTION

Lung cancer is the leading cause of cancer death worldwide [1, 2]. Due to detection at rather advanced stages, accompanied by intrinsic chemoresistance in non-small cell lung cancer (NSCLC) and rapid metastasis and therapy resistance development in small cell lung cancer (SCLC) as well as frequent relapse after surgical intervention, prognosis of this disease is very poor [3-5]. For these reasons it is clear that new strategies for rational treatment of lung cancer are urgently needed.

The fibroblast growth factor (FGF)/FGF receptor (FGFR) signaling axis is an essential system playing major roles in embryonic development and adult tissue homeostasis [6-8]. Signaling via the FGFR axis regulates gene expression important for cellular mechanisms such as proliferation, migration, survival and differentiation [8, 9]. In many tissue types, aberrant FGFR signaling has been described to promote cancer development [6, 10, 11]. The *FGFR1* gene is amplified in defined subgroups of both NSCLC and SCLC and proved to be a driving oncogene in a substantial subgroup of patients suffering from these cancer types [12, 13]. Intense research is ongoing regarding strategies to target oncogenic FGFR1 and several clinical trials to evaluate the efficacy of various FGFR inhibitors in patients with lung cancer are currently active or have already been completed [10, 14, 15].

Nintedanib is a selective small-molecule inhibitor of FGFR, vascular endothelial growth factor receptor (VEGFR) and platelet-derived growth factor receptor (PDGFR) that has recently been approved for second-line treatment after chemotherapy failure in advanced lung adenocarcinoma [15, 16]. Currently, several trials employing nintedanib are also conducted in SCLC (www.clinicaltrials.gov). Nevertheless, despite the initial success of FGFR1-targeting small molecule therapy, occurrence of acquired therapy resistance is one factor limiting the successful application of FGFR inhibitors in lung cancer [8, 17].

Data on mechanisms underlying therapy failure or resistance development with respect to small molecule FGFR inhibitors in lung cancer are limited. Therefore, this study aimed to dissect molecular factors underlying acquired FGFR inhibitor resistance in FGFR1-driven lung cancer. We have identified ATP-binding-cassette transporter B1 (ABCB1) overexpression as decisive mechanism for acquired nintedanib resistance in FGFR1-driven SCLC but not NSCLC cell models. Additionally, we demonstrate that nintedanib is a substrate of ABCB1 and, hence, this resistance mechanism needs to be considered as a factor limiting therapy response.

## RESULTS

### Selection of FGFR1-driven SCLC and NSCLC cell lines for nintedanib resistance

**Figure 1:**
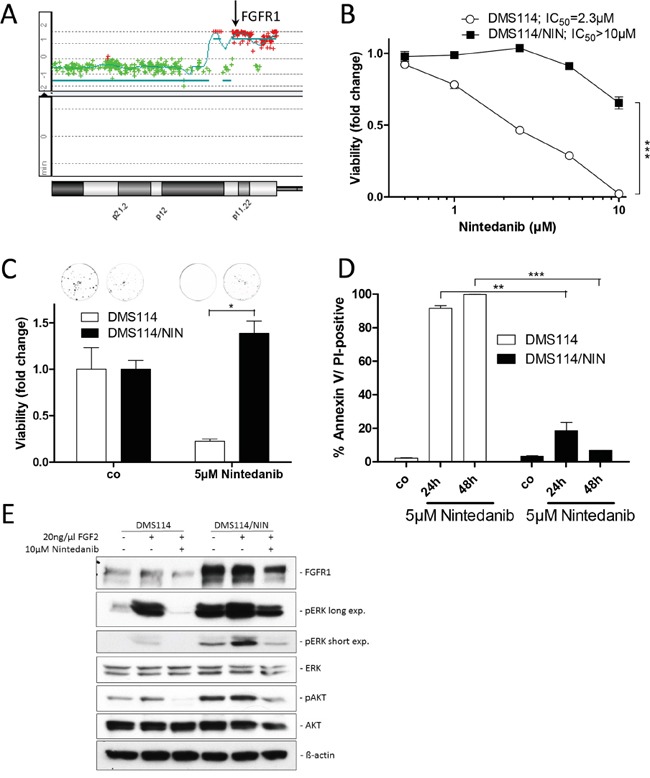
Generation of a FGFR1-driven SCLC cell line with acquired nintedanib resistance **A.** aCGH analysis was used to elucidate relative gene dose changes of DMS114 cells in comparison to normal human reference DNA. Results for the chromosome 8p arm are shown and the *FGFR1* gene locus is indicated by the *arrow*. **B.** Viability of DMS114 and DMS114/NIN cells was analyzed by MTT assay after 72 hours exposure to the indicated concentrations of nintedanib. *** p < 0.001, 2-way ANOVA, Bonferroni post-test. **C.** Impact of long-term (10d) nintedanib-exposure on clone formation capacity of DMS114 and DMS114/NIN cells was evaluated by crystal violet staining of fixed cells. Quantification was performed by densitometric measurement of stained cells using ImageJ software. Data are presented as relative values normalized to untreated controls. * p < 0.05, unpaired t-test. **D.** Apoptotic cell death induction was analyzed by Annexin V/PI-staining and FACS after 24 and 48 hours of 5μM nintedanib treatment of DMS114 and DMS114/NIN cells. ** p < 0.01 and *** p < 0.001, unpaired t-test. **E.** Expression/phosphorylation of FGFR1 and selected downstream signaling proteins in DMS114 and DMS114/NIN cells was analyzed by Western blot of total cell lysates of serum-starved cells, pre-incubated for 1h with the indicated concentration of nintedanib and stimulated for 15min with 20ng/ml FGF2. ß-actin was used as loading control.

To investigate the molecular mechanisms underlying resistance against the FGFR inhibitor nintedanib, we selected one FGFR1-driven SCLC (DMS114) and two NSCLC cell lines (NCI-H1703, NCI-H520) for acquired nintedanib resistance. All these lung cancer cell lines bear amplification of the *FGFR1* gene (shown for DMS114, Figure 1A) and have previously been shown to be hypersensitive to FGFR tyrosine kinase inhibition [13]. Exposure of cells over several months to constantly increasing nintedanib doses up to the low micromolar range resulted in pronounced acquired nintedanib resistance towards the selection drug (Figure 1B and Supplementary Figure S1). When seeded at low density, 5μM nintedanib strongly reduced clone formation capacity of DMS114 cells (75% reduction of colony formation). In contrast, at an equal concentration of nintedanib, clone formation capability of DMS114/NIN cells was not affected (Figure 1C). Also, apoptosis/cell death induction by nintedanib was significantly reduced in the subline as compared to the parental cell line, indicated by a lower percentage of cells with positive Annexin V/PI staining (Figure 1D). When stimulated for 15 minutes with the ligand FGF2, FGFR1 downstream signaling in DMS114 cells was massively activated as shown by elevated ERK and AKT phosphorylation. Preincubation of the cells with nintedanib for 1 hour completely blocked FGF2-mediated activation of FGFR1 signaling. In DMS114/NIN cells basal phosphorylation levels of FGFR1 downstream targets ERK and AKT were strongly increased and further enhanced by FGF2. In contrast to the parental cell line, nintedanib exposure of DMS114/NIN cells did not result in complete blockade of FGFR1-mediated downstream signaling (Figure 1E).

### Nintedanib-resistant subclones maintain FGFR1-signaling as oncogenic driver

**Figure 2:**
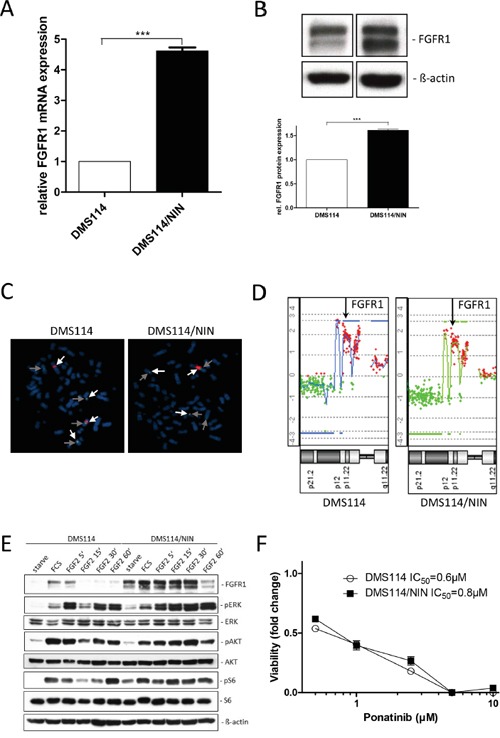
The FGFR1 signaling axis is maintained in DMS114/NIN cells upon selection for nintedanib resistance **A.** *FGFR1* mRNA expression level of DMS114/NIN cells was analyzed by quantitative RT-PCR. Data are given relative to parental cells. Expression levels were normalized to the housekeeping gene *ACTB*. *** p < 0.001, unpaired t-test. **B.** FGFR1 protein levels of DMS114 and DMS114/NIN cells analyzed by Western blot. A representative experiment (*upper panel*) is opposed to the densitometric quantification of two independent experiments using ImageJ software (*lower panel*). FGFR1 protein levels were normalized to the housekeeping gene ß-actin and the parental cells are set as 1. *** p < 0.001, unpaired t-test. **C.** Copy number evaluation of the *FGFR1* gene was performed by FISH. Representative metaphases of DMS114 and DMS114/NIN cells are shown. The *light grey arrows* indicate *FGFR1*, the *dark grey arrows* centromere 8. Chromosomes were visualized by DAPI. **D.** aCGH analysis was performed to depict relative *FGFR1* gene doses of DMS114 and DMS114/NIN cells. The respective region on chromosome 8p11 is shown and the *FGFR1* locus is indicated by *arrows*. **E.** Expression/phosphorylation of FGFR1 and selected downstream signaling proteins in DMS114 and DMS114/NIN cells was analyzed by Western blot of total cell lysates of serum-starved cells, stimulated with 10% FCS or 20ng/ml FGF2 for the indicated exposure times. ß-actin was used as loading control. **F.** Viability of DMS114 and DMS114/NIN cells was analyzed by MTT assay after 72 hours exposure to the indicated concentrations of ponatinib.

Sequencing of the kinase domain revealed no activating FGFR1 mutation in nintedanib-resistant DMS114/NIN cells (data not shown). To investigate the impact of resistance development on FGFR1 expression levels, qPCR and Western blot analyses were performed. FGFR1 expression levels were increased in the resistant DMS114/NIN subline both on mRNA (Figure 2A) and protein levels (Figure 2B). FISH analysis revealed genomic rearrangements of the *FGFR1* amplicon during selection with loss of one *FGFR1* gene copy on a derivative chromosome but gain of other strongly fluorescent *FGFR1* signals indicative for homogeneously staining regions (HSR) (arrows in Figure 2C) in DMS114/NIN cells. However, the overall *FGFR1* gene dose remained unaltered after nintedanib selection (aCGH analysis in Figure 2D). In order to test functionality of FGFR1, the two cell lines were kept under serum-free conditions as well as stimulated with FGF2. Again, basal levels of FGFR1 protein were elevated both under serum-containing and starved conditions in DMS114/NIN cells (Figure 2E). Interestingly, in the parental cell line FGF2 induced a short-term upregulation of FGFR1 (at 5 minutes exposure) followed by downregulation. In contrast, in the DMS114/NIN subline upregulation of FGFR1 persisted distinctly longer (>30 minutes) and downregulation was not seen before 60 minutes exposure. Accordingly, activation of downstream signals such as the MAPK or the PI3K/AKT pathway was stronger and more uniform in DMS114/NIN cells (Figure 2E). In contrast, in the parental cell line FGF2-mediated phosphorylation of ERK and AKT exhibited early and late peaks at 5 min and 60 min, respectively. DMS114/NIN cells exhibited no cross-resistance towards ponatinib - another FGFR inhibitor (Figure 2F). Together, this suggests that, despite constant selection pressure of nintedanib, the FGFR1 signaling axis is still maintained or even upregulated as an oncogenic driver in the DMS114/NIN subline.

### A chemical screen reveals a multidrug resistance (MDR) phenotype in DMS114/NIN cells

**Figure 3:**
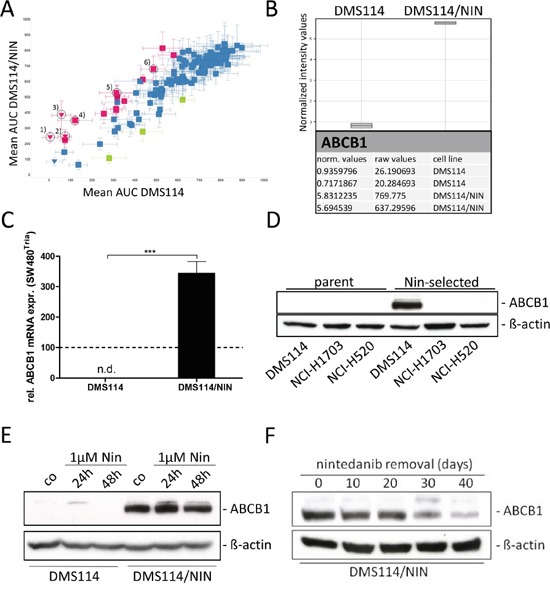
DMS114/NIN cells develop a MDR phenotype upon nintedanib selection due to upregulation of ABCB1 **A.** Sensitivities of DMS114 and DMS114/NIN cells towards different anticancer drugs were tested in a large-scale cytotoxicity screen evaluated by CellTiter Glo assay after 72 hours drug exposure. Sensitivities of DMS114 and DMS114/NIN cells are plotted as area under the curve (AUC) values determined from 8-point dose-response curves to individual drugs. *Green symbols* represent drugs less active in DMS114, *red symbols* represent drugs less active in DMS114/NIN cells. Triangles indicate compounds that were highly active in DMS114 cells with IC_50_ values below the lowest tested concentrations. Vertical error bars indicate SD of AUC of DMS114/NIN, horizontal error bars show SD of AUC of parental cells, respectively. Known ABCB1 substrate drugs are indicated by the *numbers*: *1*) carfilzomib; *2*) vinblastine; *3*) vincristine; *4*) mitoxantrone; *5*) docetaxel; *6*) CUDC-101; **B.** Whole-genome gene expression patterns of DMS114 and DMS114/NIN cells were established by microarray analysis and data were processed using GeneSpring software (Agilent Technologies). Raw and normalized mRNA expression values are depicted for *ABCB1*. **C.** *ABCB1* mRNA expression was analyzed by quantitative RT-PCR and data are given normalized to *ACTB* mRNA expression. *** p<0.001, unpaired t-test. ABCB1-overexpressing SW480^Tria^ cells served as positive control and were set as 100 (dashed line). n.d., not detected; **D.** ABCB1 protein expression levels of DMS114 (SCLC), NCI-H1703 and NCI-H520 (both NSCLC SCC) and its respective nintedanib-selected sublines were determined by Western blot. ß-actin was used as loading control. **E.** Impact of short-term (24 and 48 hours) nintedanib exposure on ABCB1 expression in DMS114 and DMS114/NIN cells was analyzed by Western blot analysis. ß-actin was used as loading control. **F.** Impact of removal of nintedanib selection pressure on ABCB1 expression levels in DMS114/NIN cells was analyzed by Western blot analysis over 40 days. ß-actin was used as loading control.

To investigate the impact of acquired nintedanib resistance on the sensitivity against other cancer drugs, a high-throughput screen employing a library of approved and experimental compounds was performed. This screen revealed distinct cross-resistance of the DMS114/NIN subline against several anticancer agents (red symbols in Figure 3A) including e.g. vincristine, vinblastine, mitoxantrone, docetaxel, carfilzomib as well as the small molecule EGFR inhibitor CUDC-101. Noteworthy, all these compounds are known substrates of ABC transporter drug efflux pumps conferring a multidrug resistance (MDR) phenotype.

### Transcriptional upregulation of ABCB1 underlies the MDR phenotype of DMS114/NIN cells

These results prompted us to perform whole-genome gene expression arrays to detect mRNA expression differences of genes potentially causing the observed MDR phenotype. Regarding all ABC transporter efflux pumps, only expression of *ABCB1* mRNA was distinctly enhanced in DMS114/NIN cells (Figure 3B), which is known to affect all cross-resistant drugs identified in the drug screen (Figure 3A). Significantly enhanced expression of this gene could be confirmed by qPCR (Figure 3C). Accordingly, high ABCB1 protein expression was confirmed by Western blot (Figure 3D). In contrast, neither *ABCC1* nor *ABCG2* - the two other highly important resistance-mediating ABC transporter genes – were expressed at detectable levels in DMS114/NIN cells (data not shown). To further elucidate whether ABCB1 upregulation upon nintedanib selection is restricted to SCLC, we tested our two additional FGFR1-driven NSCLC cell lines (NCI-H1703/NIN, NCI-H520/NIN) with acquired nintedanib resistance. Despite identical conditions of *in vitro* selection, overexpression of ABCB1 was only observed in DMS114/NIN cells (Figure 3D).

Next, we investigated whether ABCB1 expression in response to nintedanib is a consequence of immediate stress response. However, exposure of the parental cell line to 1μM nintedanib up to 48 hours did not induce detectable levels of ABCB1 expression (Figure 3E). Removal of nintedanib selection pressure for up to 40 days resulted in gradual loss of ABCB1 expression in DMS114/NIN cells (Figure 3F), leading to a significant attenuation of resistance in the revertant cell line towards nintedanib and vincristine. Nevertheless, resistance remained highly significant after this period without selection pressure (Supplementary Figure S2A and S2B).

### Inhibition of ABCB1 resensitizes DMS114/NIN cells to nintedanib as well as other MDR substrate drugs

**Figure 4:**
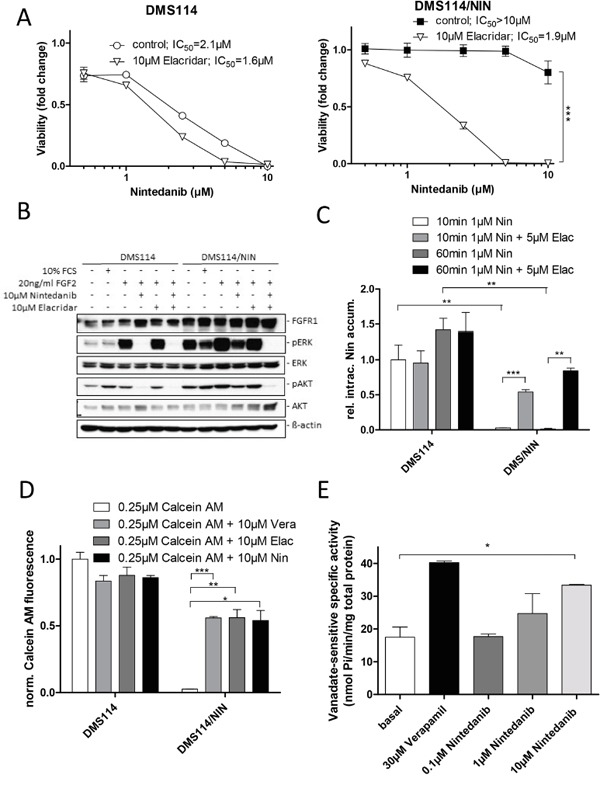
Nintedanib is a substrate of ABCB1-mediated drug efflux **A.** Impact of the ABCB1 modulator elacridar on the anticancer activity of nintedanib in DMS114 and DMS114/NIN cells was measured by MTT assay after 72 hours drug exposure. *** p<0.001, 2-way ANOVA, Bonferroni post-test. **B.** The impact of the ABCB1 modulator elacridar on nintedanib-mediated inhibition of FGFR1 downstream target phosphorylation in DMS114 and DMS114/NIN cells was analyzed by Western blot. Serum-starved cells were treated with indicated concentrations of nintedanib or elacridar for 1h and stimulated with 10% FCS or 20ng/ml FGF2 for 15min. ß-actin was used as loading control. **C.** Intracellular levels of nintedanib in DMS114 and DMS114/NIN cells after 10 or 60min exposure to 1μM of the drug in the presence or absence of 5μM elacridar was determined by LC-MS. ** p < 0.01, *** p < 0.001, unpaired t-test. **D.** Impact of nintedanib on intracellular accumulation of the fluorescent ABCB1 substrate calcein. DMS114 and DMS114/NIN cells were incubated with calcein AM for 15min in the presence or absence of 10μM nintedanib. The ABCB1 modulators verapamil and elacridar served as positive controls. Intracellular calcein fluorescence was measured by FACS and analyzed by FlowJo software. * p < 0.05, ** p < 0.01, *** p < 0.001, unpaired t-test. **E.** Impact of nintedanib on the ABCB1 ATPase function was determined using ABCB1-enriched Sf9 cells. Crude Sf9 cell membrane vesicles were exposed to indicated concentrations of nintedanib in presence or absence of ATPase inhibitor vanadate. ATPase activation was measured through colorimetric quantification of liberation of inorganic phosphate. ATPase activation by 30μM verapamil served as positive control. * p < 0.05, unpaired t-test. Vera= verapamil, Elac= elacridar, Nin= nintedanib;

Next, we investigated whether pharmacological inhibition of ABCB1 restores sensitivity of DMS114/NIN cells towards nintedanib as well as known ABCB1 substrate drugs. Presence of the third generation ABCB1 inhibitor elacridar (10μM), itself not influencing cell viability, almost completely abrogated acquired nintedanib resistance of DMS114/NIN cells (Figure 4A and Supplementary Figure S3A). Elacridar is known to inhibit also the ABCG2 efflux pump. However, microarray data and Western blotting suggested very low and unaltered ABCG2 expression levels in DMS114/NIN cells as compared to the parental cell line (data not shown). This suggests that ABCB1 activity negatively interferes with nintedanib cytotoxicity, possibly by direct efflux. Accordingly, transfection of parental cells with an ABCB1-encoding expression vector led to a significantly reduced sensitivity towards nintedanib (Supplementary Figure S3B, S3C). DMS114/NIN cells also exhibited pronounced cross-resistance against the investigated ABCB1 substrates vincristine, vinblastine and doxorubicin (Supplementary Figure S4A, S4B and S4C, respectively). Coincubation with elacridar resensitized the subline to the tested drugs, while having no effect on the cytotoxicity in the parental cells (Supplementary Figure S4). With regard to FGFR1-downstream signaling in response to FGF2, cotreatment with elacridar led to restoration of ERK and AKT phosphorylation inhibition by nintedanib in the DMS114/NIN subline (Figure 4B). Accordingly, intracellular levels of nintedanib were strongly reduced in DMS114/NIN as compared to DMS114 cells after short-term exposure (Figure 4C). However, in the presence of elacridar, intracellular nintedanib levels of DMS114/NIN, but not the parental cells were distinctly increased, proving that nintedanib is a substrate of ABCB1-mediated drug efflux.

Additionally, we tested whether nintedanib is an inhibitor of ABCB1 using the fluorescent dye Rh123 as substrate (Supplementary Figure S5A). As expected, accumulation of Rh123 was significantly reduced in DMS114/NIN as compared to DMS114 cells. Nintedanib moderately but significantly enhanced Rh123 accumulation in the selected subline, however, to a distinctly lesser extent than did elacridar. This indicates that nintedanib is a weakly competitive inhibitor of ABCB1-mediated Rh123 export. In contrast, ABCB1-driven efflux of calcein AM was inhibited with similar potency by elacridar, verapamil and nintedanib, suggesting a preferential binding of nintedanib to the calcein AM binding site of ABCB1 (Figure 4D). Moreover, nintedanib dose-dependently stimulated the ATPase activity of ABCB1 in Sf9 crude membrane extracts with a maximum effect at the highest tested dose (10μM), where nintedanib exhibited a potency almost comparable with the positive control verapamil (Figure 4E) [18].

### Selection of DMS114 cells for resistance against another FGFR tyrosine kinase inhibitor (TKI) also leads to upregulation of ABCB1

**Figure 5:**
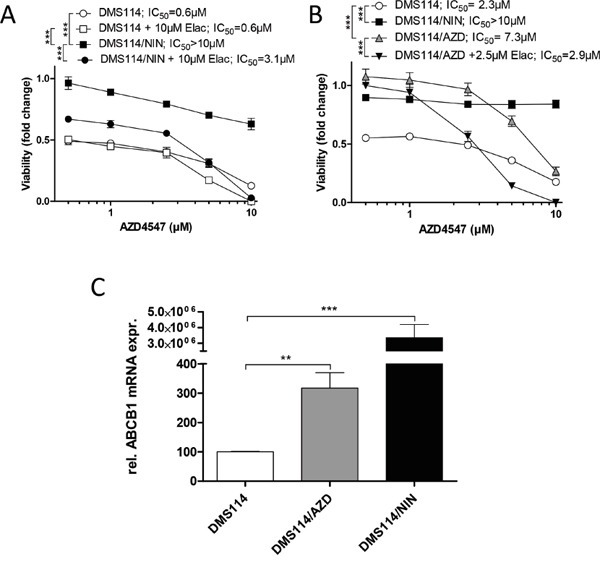
Selection of DMS114 cells for acquired AZD4547 resistance also mediates *ABCB1* upregulation **A.** Impact of the ABCB1 modulator elacridar on the anticancer activity of AZD4547 in DMS114 and DMS114/NIN cells was measured by MTT assay after 72 hours drug exposure. *** p<0.001, 2-way ANOVA, Bonferroni post-test. **B.** Viability of DMS114, DMS114/NIN and DMS114/AZD cells was analyzed by MTT assay after 72 hours exposure to the indicated concentrations of AZD4547. *** p < 0.001, 2-way ANOVA, Bonferroni post-test. **C.** *ABCB1* mRNA expression was analyzed by quantitative RT-PCR and data are given normalized to *ACTB* mRNA expression. ** p < 0.01, *** p<0.001, unpaired t-test.

We were interested whether selection of DMS114 for acquired resistance against other FGFR inhibitors susceptible to ABCB1-mediated drug efflux might also result in ABCB1 upregulation. We, thus, first evaluated the sensitivities of DMS114/NIN cells and their parental cell line towards other FGFR TKIs. Interestingly, DMS114/NIN cells exhibited marked resistance towards the small molecule inhibitor AZD4547, which could be reversed by coincubation with elacridar. This suggests that AZD4547 is a substrate for ABCB1-mediated efflux (Figure 5A). Based on these findings, we established a subline of DMS114 cells with acquired resistance towards AZD4547 (DMS114/AZD; Figure 5B). After several weeks of constant drug exposure, qPCR analysis revealed significantly elevated *ABCB1* expression levels in DMS114/AZD cells (Figure 5C). Elacridar significantly diminished AZD4547 resistance of DMS114/AZD cells (Figure 5B). Together, these data suggest that chronic exposure of DMS114 cells to ABCB1 substrate FGFR inhibitors generally leads to ABCB1-mediated resistance acquisition.

### Nintedanib resensitizes an independent MDR model to ABCB1 substrate drugs

**Figure 6:**
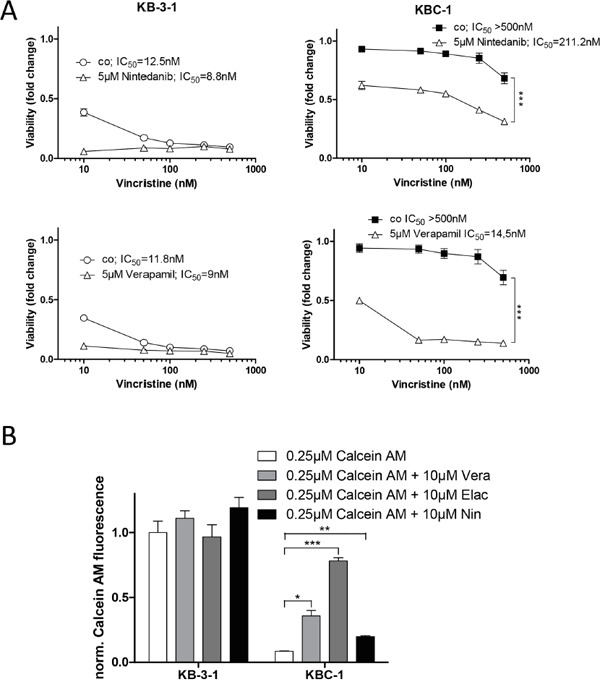
Nintedanib resensitizes multidrug resistant KBC-1 cells to ABCB1 substrate drugs **A.** Viability of KB-3-1 and KBC-1 was analyzed by MTT assay after 72 hours exposure to vincristine in the presence or absence of 5μM nintedanib. Verapamil (*lower panels*) was used as positive control for ABCB1 modulation. *** p<0.001, 2-way ANOVA, Bonferroni post-test. **B.** Impact of nintedanib on intracellular accumulation of the fluorescent ABCB1 substrate calcein was determined. KB-3-1 and KBC-1 cells were incubated with calcein AM for 15 minutes in the presence or absence of 10μM nintedanib. Intracellular calcein fluorescence was measured by FACS and analyzed by FlowJo software. The ABCB1 modulators verapamil and elacridar served as positive controls. * p < 0.05, ** p < 0.01, *** p < 0.001, unpaired t-test. Vera= verapamil, Elac= elacridar, Nin= nintedanib;

We further raised the question whether nintedanib could also reverse ABCB1-mediated drug resistance in an independent MDR model. Therefore, we tested the cervix carcinoma cell line KB-3-1 and its ABCB1-overexpressing sublines KBC-1 and KB-V1 [19] for sensitivity against vincristine (Figure 6A) and vinblastine (Supplementary Figure S5B) in absence and presence of nintedanib. While having only minor effects on the cytotoxicity in KB-3-1, 5μM nintedanib partially restored sensitivity towards both vincristine and vinblastine in KBC-1 cells. Accordingly, 10μM nintedanib moderately but significantly increased intracellular accumulation of calcein in KBC-1 cells (Figure 6B). This effect was also observed for accumulation of Rh123 (Supplementary Figure S6A). In addition, inhibition of ABCB1 by elacridar resensitized KBC-1 and KB-V1 cells against high doses of nintedanib (Supplementary Figure S6B and S6C).

### Lack of *ABCB1* gene amplification and promoter methylation changes in DMS114/NIN cells

**Figure 7:**
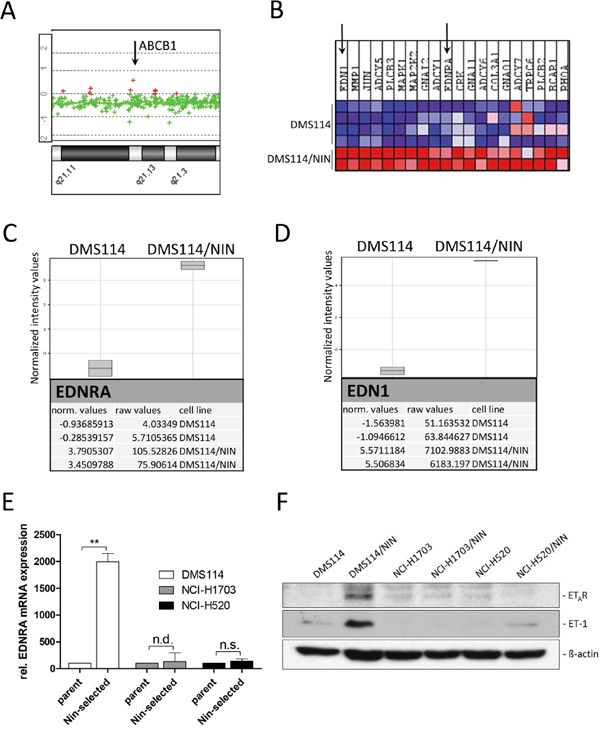
The endothelin-A receptor (ET_A_R) signaling axis contributes to ABCB1 upregulation in DMS114/NIN cells **A.** Relative *ABCB1* gene dose of DMS114/NIN as compared to their parental cell line was evaluated by indirect aCGH analysis. A part of chromosomal region 7q21 is shown and the *ABCB1* gene locus depicted by the *arrow*. **B.** GSEA revealed significant enrichment of the pathway interaction database-derived GO term ‘endothelin-pathway’ for upregulation in DMS114/NIN as compared to DMS114 cells. The 20 most upregulated genes are depicted as heatmap and identified endothelin-1 (*EDN1*/ET-1) and endothelin-A receptor (*EDNRA*/ET_A_R) to be among the top upregulated genes in DMS114/NIN cells (indicated by *arrows)*. **C, D.** Raw and normalized mRNA expression values of the endothelin signaling family members revealed selective upregulation of genes encoding endothelin-A receptor (*EDNRA*) (C) and endothelin-1 (*EDN1*) (D). Data were analyzed using GeneSpring (Agilent Technologies) and GSEA softwares. **E.** mRNA expression levels of ET_A_R (*EDNRA)* in DMS114, NCI-H1703, NCI-H520 cells and their respective sublines were determined by quantitative RT-PCR. ** p<0.01, one-sample t test; n.s., not significant; n.d., not detected before qPCR cycle 36; **F.** ET_A_R and ET-1 protein levels in DMS114, NCI-H1703 and NCI-H520 cells and their respective sublines were analyzed by Western blot. ß-actin was used as loading control.

Genetic amplification of the *ABCB1* locus is frequently observed in MDR cell lines selected against cytotoxic anticancer drugs [20]. However, we found no *ABCB1* gene dose alteration in DMS114/NIN cells by indirect aCGH (Figure 7A). Decreased methylation of seven CpG dinucleotides in the *ABCB1* promoter downstream the transcription start site (from +524 to +587) was previously found to be associated with enhanced gene expression [21]. However, DNA methylation of all these CpG dinucleotides was below the pyrosequencing limit of quantification in both the parental DMS114 cells and the resistant subline (data not shown). This suggests that neither gene amplification nor epigenetic regulation by altered promoter methylation cause upregulation of *ABCB1* gene expression in DMS114/NIN cells.

### Endothelin-A receptor signaling contributes to ABCB1 overexpression in DMS114/NIN cells

**Figure 8:**
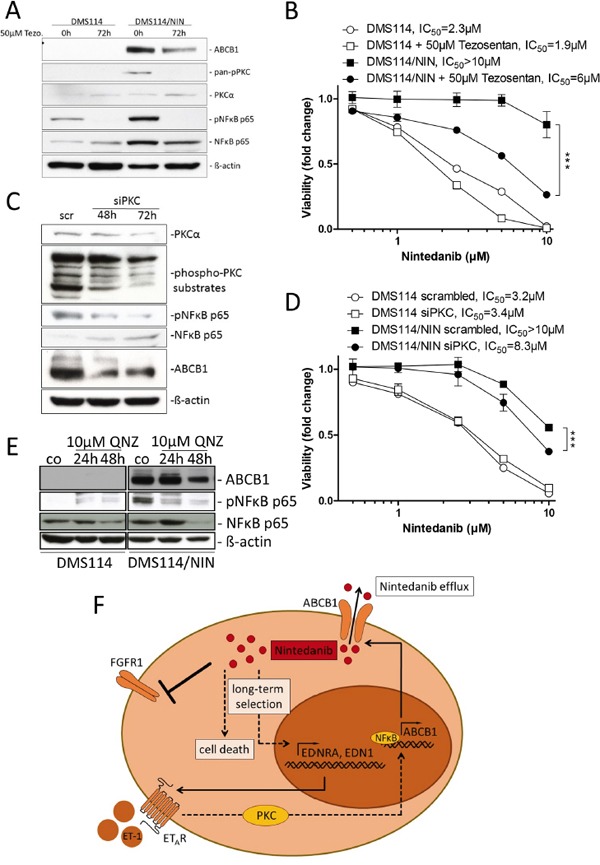
Inhibition of the PKC/NFκB signaling axis downregulates ABCB1 and resensitizes DMS114/NIN cells towards nintedanib **A.** Impact of ET_A_R blockade by its antagonist tezosentan on expression and phosphorylation levels of indicated proteins in DMS114 and DMS114/NIN cells was analyzed by Western blot. ß-actin was used as loading control. **B.** Impact of ET_A_R blockade on viability of nintedanib-treated DMS114 and DMS114/NIN cells was measured by MTT assay. Cells were pretreated for 72 hours with 50μM tezosentan. Viability was measured after another 72 hours exposure to nintedanib in combination with tezosentan. *** p<0.0001, 2-way ANOVA, Bonferroni post-test. **C, D.** Transient knockdown of PKC was performed in DMS114 and DMS114/NIN cells by siRNA transfection. C. Impact of 50nM siPKC knockdown in DMS114/NIN cells on PKCα protein levels and phosphorylation of indicated PKC downstream targets was analyzed by Western blot 48 and 72 hours post-transfection. 50nM non-targeted siRNA (scr) served as transfection control. D. Impact of transient PKC knockdown on viability of DMS114 and DMS114/NIN cells after 72 hours exposure to nintedanib was analyzed by MTT assay. Cells pretreated with 50nM scrambled siRNA served as transfection control. **E.** Impact of NFκB inhibition by QNZ for 24 and 48 hours on ABCB1 expression in DMS114 and DMS114/NIN cells was determined by Western blot analysis. ß-actin was used as loading control. *** p<0.001, 2-way ANOVA, Bonferroni post-test. **F.** Schematic representation showing the mechanism of acquired resistance of DMS114 cells towards nintedanib.

In search of transcriptional alterations potentially underlying *ABCB1* upregulation, gene expression data of DMS114 and DMS114/NIN cells were compared by GSEA. Noteworthy, several enriched gene ontology (GO) terms within the cellular pathway-, protein interaction as well as the reactome-databases concerned G-protein coupled receptor signaling. These GO terms included the endothelin pathway, GPCR-ligand binding, peptide ligand binding receptors as well as rhodopsin-like receptors, all containing members of the endothelin signaling family (Supplementary Figure S7A-S7D). Subsequent analysis on the single gene level revealed endothelin-1 (*EDN1*/ET-1) and the respective endothelin-A receptor (*EDNRA*/ET_A_R) to be among the top upregulated genes in DMS114/NIN cells (Figure 7B-7D). Endothelin signaling has been reported to activate ABCB1 expression in brain capillary endothelial cells via endothelin receptors A and B via activation of PKC and subsequently NFκB [22]. Transcriptional upregulation of *EDNRA* in DMS114/NIN cells was validated by qRT-PCR (Figure 7E) and elevated levels of both ET-1 and ET_A_R were also confirmed on protein level (Figure 7F). Interestingly, nintedanib-selected sublines of NCI-H1703 and NCI-H520 cells, both lacking ABCB1 overexpression, also exhibited low and unaltered ET_A_R and ET-1 expression levels as compared to their parental cell lines (Figure 7E and 7F). Accordingly, blockade of this receptor with its specific antagonist tezosentan for 72 hours led to a distinct decrease in ABCB1 protein levels in DMS114/NIN cells (Figure 8A) supporting a role in transcriptional *ABCB1* activation. Interestingly, both ET_A_R downstream effectors PKC and NFκB p65 tended to be hyperphosphorylated and, hence, hyperactivated in DMS114/NIN as compared to DMS114 cells. ET_A_R blockade by tezosentan abolished phosphorylation of both PKC isotypes and NFκB p65 in the nintedanib-resistant subline (Figure 8A). Antagonism of ET_A_R by tezosentan also led to a significant re-sensitization of DMS114/NIN cells towards nintedanib, while having only a minor effect in the parental cell line (Figure 8B). In line with these findings, partial PKC knockdown by siRNA resulted in decreased levels of ABCB1, pNFκB p65 and a significantly increased cytotoxicity of nintedanib in DMS114/NIN cells (Figure 8C and 8D, respectively). A comparably increased sensitivity of DMS114/NIN cells towards nintedanib and vincristine was also observed upon co-incubation with the pharmacological PKC inhibitor BIMI (Supplementary Figure S8A and S8B, respectively). Accordingly, also blockade of NFκB by the selective inhibitor QNZ for 48 hours resulted in downregulation of ABCB1 (Figure 8E). In summary, this suggests that transcriptional upregulation of *ABCB1* in DMS114/NIN cells upon selection for nintedanib resistance is induced at least in part by an ET_A_R-mediated activation of the PKC-NFκB signaling pathway (summarized in Figure 8F).

## DISCUSSION

The *FGFR1* gene is amplified in lung cancer at varying frequencies (depending on the histological subtype between 5.6% and 19% for SCLC and NSCLC SCC, respectively) and overexpression of FGFR1 was shown to be a driving oncogenic factor in subgroups across all lung cancer subtypes [23-26]. Nintedanib, a small-molecule FGFR/PDGFR/VEGFR TKI, demonstrated efficacy in clinical trials against lung cancer and is approved for second-line treatment of lung adenocarcinoma [15]. Additionally, it is currently being evaluated clinically in SCLC [5]. As observed for all targeted anticancer agents, treatment of lung cancer patients with nintedanib is not curative and leads to disease recurrence due to acquired resistance development [15, 16]. Therefore, we were interested in elucidating the molecular mechanisms underlying acquired insensitivity of FGFR1-driven SCLC and NSCLC against nintedanib. Out of the three nintedanib-refractory cell models generated, the SCLC cell line DMS114/NIN was characterized by a cross-resistance pattern in an extended drug screen with insensitivity against several chemotherapeutics but also TKIs. This phenotype resembles a phenomenon known as multidrug resistance (MDR) which is frequently based on the overexpression of pleiotropic efflux pumps of the ATP-binding-cassette (ABC) transporter family (particularly of ABCB1, ABCG2 and ABCC1) [27-29]. Our subsequent analyses confirmed that massive overexpression of ABCB1 is underlying the MDR phenotype and nintedanib resistance of DMS114/NIN cells.

ABC transporters are transmembrane proteins which translocate a wide range of substances across cellular membranes [20]. One of their most important functions represents the protection of tissues from toxins and xenobiotics. Different ABC transporter subtypes are expressed on various non-malignant tissue types functioning as protective barriers in the body, including amongst others the endothelial layer of the cerebral microvasculature and the epithelial lining of the intestinal tract [30]. Substrates of ABC-mediated efflux include a wide range of biological compounds but also chemotherapeutics and targeted anticancer agents such as EGFR inhibitors [31, 32]. However, knowledge of the impact of ABC transporters on resistance against FGFR TKIs is limited. So far, only reversal of ABCB1-mediated MDR by the pan-FGFR/VEGFR inhibitor PD173074 and, in agreement with our here presented data, also nintedanib has been described [33, 34]. In the current study we demonstrate that high level expression of ABCB1 is underlying acquired nintedanib resistance of the FGFR1-driven SCLC cell line DMS114 but not of the two NSCLC cell lines NCI-H1703 and NCI-H520. Furthermore, we proved that nintedanib is a direct substrate of ABCB1. Importantly, nintedanib was also able to restore sensitivity of DMS114/NIN cells, colchicine-selected KBC-1 [35] and vinblastine-selected KB-V1 cells towards other ABCB1 substrate compounds leading to synergistic effects in combination experiments especially in a FGFR-driven background. It is of interest why the upregulation of ABCB1 expression in response to nintedanib treatment occurred exclusively in the SCLC and not in the two NSCLC cell lines. The marked propensity of SCLC to develop chemoresistance in response to anticancer therapy has been described to be -at least partly-mediated by overexpression of ABC transporters [36]. However, upregulation of ABCB1 expression in lung cancer does not appear to be exclusive for SCLC, as it has also been observed in NSCLC following chemotherapy [37]. With respect to targeted small molecule inhibitors, no connection between lung cancer subtypes and ABCB1 induction has been described. So far, only overexpression of ABCG2 in healthy bronchiolar progenitor cells has been observed to mediate efficient drug efflux. However, at present there is no data on ABCB1 expression levels in pulmonary neuroendocrine cells, the non-malignant SCLC counterpart. Therefore, it will be interesting to elucidate whether ABCB1 overexpression during selection of DMS114 for acquired resistance reflects a SCLC cell type-specific response to nintedanib treatment.

While ABCB1-mediated resistance acquisition is frequently observed as a consequence of the selection against chemotherapeutic agents, the examples regarding TKI resistance are comparably sparse. Reports started with ABCB1 and ABCG2 overexpression in Philadelphia chromosome-positive chronic myeloid leukemia cells as a consequence of imatinib selection [38]. Recently, also acquisition of EGFR and MET inhibitor resistance was occasionally connected with ABCB1 overexpression and concerned afatinib and erlotinib as well as PHA-665752, respectively [39, 40]. In the case of afatinib and PHA-665752 resistance, upregulation of ABCB1 expression was connected with selection for a cancer stem cell phenotype and an epithelial-to-mesenchymal transition (EMT) switch [41]. In our study, however, selection of a DMS114 cell subclone with enhanced stemness during nintedanib selection seems unlikely. Gene set enrichment analysis (GSEA) did not indicate any upregulation of stem cell markers. Furthermore, DMS114/NIN cells exhibited rather reduced than enhanced growth as three-dimensional spheroids *in vitro* and as subcutaneous xenografts *in vivo* (data not shown).

In search of factors underlying the massive ABCB1 upregulation, GSEA suggested hyperactivation of several G-coupled protein receptor pathways in DMS114/NIN cells. At a closer look, it turned out that all of these GO terms contained the soluble polypeptide ligand ET-1 and its cognate G-protein-coupled receptor ET_A_R. Indeed, the ET-1/ET_A_R signaling axis, with its downstream effectors PKC and NFκB, was hyperactivated in DMS114/NIN cells and significantly contributed to acquired nintedanib resistance. Hence, antagonism of ET_A_R by tezosentan led to decreased levels of ABCB1 protein accompanied by decreased PKC and NFκB phosphorylation and, importantly, to a significant re-sensitization of DMS114/NIN cells towards nintedanib. The endothelin signaling axis represents a promising therapy target based on various protumorigenic effects. These include, amongst others, epithelial-to-mesenchymal transition (EMT) and, importantly, chemoresistance by induction of stemness features [42]. However, neither enrichment of an EMT nor a stem cell signature was suggested by our bioinformatic analyses in DMS114/NIN cells. Interestingly, Bauer and colleagues suggested an essential contribution of the ET-1/ET_A_R signaling axis to constitutive ABCB1 expression in brain capillary endothelial cells as a functional component of the blood-brain-barrier [22]. The authors additionally found ABCB1 expression to be induced by ET_A_R through activation of PKC and NFκB downstream signaling. In the light of our here presented data, this implies that cancer cells can utilize physiological signal circuits, as in this case ABCB1 activation by ET-1/ET_A_R signaling in endothelial cells, to survive systemic treatment options. Activation of MDR by this particular physiological brain protection mechanism has not been reported in the malignant background so far. One might hypothesize that ET_A_R-mediated ABCB1 activation is a consequence of the specific nintedanib target profile including major players in endothelial cell physiology like FGFR and VEGFR. Indeed, in a study by Huang et al., selection against the VEGFR/PDGFR angiokinase inhibitor sunitinib led to induction of ABCB1 expression in transformed HMEC-1 endothelial cells [43]. Alternatively, based on the dominance of gene amplification and epigenetic regulation in ABCB1-mediated MDR, the impact of such more subtle physiological mechanisms might have been overlooked so far in the malignant background. Nevertheless, upregulation of both ET_A_R/ET-1 and ABCB1 was not observed in two FGFR-driven NSCLC cell lines, suggesting a possible impact of the SCLC histology.

Concerning the clinics, the fact that nintedanib is an ABCB1 substrate might impair its efficacy as a single treatment not only in lung cancer, but also in other cancer types. For instance, high intrinsic ABCB1 expression in clear cell renal carcinoma might render nintedanib treatment ineffective [44]. Additionally, the constitutive expression of ABC transporters at the blood brain barrier might limit applicability of nintedanib in cancers of the central nervous system [45]. On the contrary, nintedanib might function as a MDR reverser within combination treatment schemes, thus impeding chemotherapy-induced drug resistance. Moreover, combination approaches with ET_A_R antagonists or a switch to non-ABCB1 substrate FGFR inhibitors represent innovative strategies to manage nintedanib resistance in lung cancer.

## MATERIALS AND METHODS

### Cell culture

The human SCLC cell line DMS114 and the NSCLC cell lines NCI-H1703 and NCI-H520 were obtained from American Type Culture Collection (Manassas, VA). The human cervix carcinoma cell line KB-3-1, its colchicine-resistant, ABCB1-overexpressing subline KBC-1 and its vinblastine-resistant, ABCB1-overexpressing subline KB-V1 were a generous gift from Dr. Shen, Bethesda, USA [35]. KB-V1 cells were grown in DMEM, all other cell lines were cultured in RPMI 1640 media supplemented with 10% fetal calf serum. All cell lines were authenticated by array comparative genomic hybridization and regularly checked for *Mycoplasma* contamination (Mycoplasma Stain kit, Sigma, St. Luis, USA).

### Drugs and chemicals

Nintedanib, ponatinib, AZD4547, PD173074 and QNZ were purchased from Selleckchem (Munich, Germany), bisindolylmaleimide I (BIMI) from Cayman Chemical (Michigan, USA), FGF2 from Eubio (Peprotech, Rocky Hill, USA), verapamil from Abbott (Illinois, USA), Calcein AM from eBioscience (San Diego, USA), vanadate, elacridar, vincristine, vinblastine, doxorubicin from Sigma. Tezosentan was obtained from Actelion Pharm, Allschwil, Switzerland.

### Selection of DMS114 cells for acquired nintedanib and AZD4547 resistance

DMS114, NCI-H1703 and NCI-H520 sublines were generated by constant *in vitro* exposure to submicromolar doses of nintedanib. In regular intervals, drug dose was continuously elevated, reaching a maximum dose of 10μM after approximately 1 year. Nintedanib-resistant sublines were designated DMS114/NIN, NCI-H1703/NIN and NCI-H520/NIN. The AZD4547-selected subline of DMS114 cells was termed DMS114/AZD. Resistance levels were constantly monitored by cell viability assay (MTT).

### Array comparative genomic hybridization (aCGH)

aCGH was performed on 4×44K oligonucleotide-based microarrays (Agilent, Santa Clara, USA) as previously described [46]. DNA labeling and hybridization was performed according to the manufacturer's instructions. For direct aCGH, DMS114 was compared to normal human reference DNA, for indirect aCGH, DMS114/NIN was compared directly to its parental line DMS114.

### Cell viability assay

To determine cell viability, 3×10^4^ cells were seeded in 96-well plates and allowed to adhere for 24 hours. Cells were exposed to test compounds and after 72 hours, cell survival was determined by the 3-(4,5-dimethylthiazol-2-yl)-2,5-diphenyltetrazolium bromide (MTT)-based vitality assay (EZ4U, Biomedica, Vienna, Austria) according to the manufacturer's instructions. Dose-response curves were generated by GraphPad Prism software and cytotoxicity levels were expressed as IC_50_ values, indicating drug concentrations resulting in a 50% reduction of cell number in comparison to untreated controls.

### Clone formation assay

To determine the ability of single cells for clonal expansion at low density in the presence of nintedanib, 5×10^2^ cells were seeded in 24-well plates and were allowed to adhere for 24 hours. Clone formation under exposure with indicated drug concentrations was followed for 10 days. Cells were fixed with ice-cold methanol and stained with crystal violet. Degree of clonal expansion was determined by colorimetric quantification of stained cells using ImageJ software.

### Annexin V/propidium iodide (PI) staining and FACS analysis

To determine apoptosis (Annexin V, BD Biosciences, Franklin Lakes, USA) and cell death (PI, Sigma, USA) induction, cells were treated with indicated concentrations of nintedanib for 24 and 48 hours. Cells were trypsinized and stained with APC-labeled Annexin V and PI and subjected to FACS analysis (FACScalibur, Becton Dickinson, Palo Alto, USA). Extent of apoptosis/cell death induction was analyzed by FlowJo software.

### RNA isolation and real-time PCR

Total RNA from cell lysates was isolated by Trizol reagent (Life Technologies, Carlsbad, USA). mRNA was reverse transcribed (Thermo Fisher Scientific, Waltham, USA) and resulting cDNA was used to perform TaqMan quantitative real-time PCR as described previously [47] using following primers: *FGFR1* sense: 5′-CCTCTTCTGGGCTGTGCT-3′, *FGFR1* antisense: 5′-CGGGCATACGGTTTGGTT-3′, *ABCB1* sense: 5′-CCCATCATTGCAATAGCAGG-3′. ABCB1 antisense: 5′-GTTCAAACTTCTGCTCCTGA-3′, *EDNRA* sense: 5′-GGGATCACCGTCCTCAACCT-3′, *EDNRA* antisense: 5′-CAGGAATGGCCAGGATAAAGG-3′, *ACTB* sense: 5′-GGATGCAGAAGGAGATCACTG-3′, *ACTB* antisense: 5′-CGATCCACACGGAGTACTTG-3′. *ACTB* served as internal control.

### Western blot analysis

Protein extracts from total cell lysates were prepared, separated by SDS-PAGE and blotted onto polyvinylidene difluoride membranes (PVDF, Fisher Scientific). FGFR1, phospho-ERK1/2 (Thr202/Tyr204), ERK1/2, AKT, phospho-AKT, PKCα, pan-phospho-PKC, NFκB p65, pNFκB p65 and pPKC-substrate antibody were all purchased from Cell Signaling Technology (Danvers, USA). ß-actin monoclonal mouse AC-15 was obtained from Sigma and ABCB1 (C219) from BioLegend (San Diego, USA). Horseradish peroxidase-conjugated secondary antibodies (Santa Cruz Biotech, Dallas, USA) were used as 1:10.000 working dilutions.

### Metaphase chromosome preparation and fluorescent *in situ* hybridization (FISH)

Metaphase chromosome spreads of DMS114 and DMS114/NIN cells were prepared by standard techniques. Chromosomes were co-denatured and hybridized with fluorophore-labeled FISH-probes (Abbott) targeting the *FGFR1* locus as well as centromere chromosome 8 and subjected to fluorescence microscopic analysis.

### Anticancer compound screen

A large-scale drug screen was performed in 384-well plates on a cell::explorer platform (PerkinElmer, Waltham, Massachusetts, USA). In a pre-screen, DMS114, NCI-H1703, NCI-H520 cells and their respective nintedanib-selected sublines were tested against a set of 549 compounds at a final concentration of 20μM (0.1% DMSO) measured in duplicates. 10μM vincristine served as positive control and was set to 0%. 0.1% DMSO served as negative control and was set to 100%. Compounds were transferred into drug plates by acoustic droplet ejection using an Echo 520 liquid handler (LABCYTE, Sunnyvale, CA, USA). Cells were seeded onto drug plates at a density of 1×10^3^ cells/well. After 72h, cell viability was determined by CellTiter Glo Assay (Promega, Madison, USA) according to manufacturer's instructions on a EnVision plate reader (PerkinElmer). Data analysis was performed by calculating a percentage of control to normalize for variability across different plates. Signal intensities for the negative control DMSO wells were set to 0%, wells containing the positive control vincristine were set to 100% for each plate individually. Hits were defined as compounds that gave > 50% inhibition compared to the DMSO controls. The pre-screen yielded 136 positive hits. In a subsequent screen, differences in sensitivity of the cell lines and their respective sublines towards the identified drugs were analyzed in 8-point dose-response curves (3-fold dilutions typically starting at 13.5μM, 0.135% DMSO, 13.5μM Bortezomib served as positive control). A mean Z-factor of 0.277756 was calculated over all plates [48]. Areas under the curve (AuC) were calculated as a cumulative measure of compound potency by doing the sum of the mean of subsequent concentration points. Hits were defined as a 50% difference in viability at one concentration or a similar cumulative effect over all concentrations and 1 standard deviation difference between the two means. According to these criteria, 39 compounds were identified as differentially cytotoxic in at least one of the three cell line pairs.

### Whole genome gene expression array

Whole genome gene expression arrays were performed on 4×44K whole genome oligonucleotide-based gene expression arrays (Agilent, Santa Clara, USA) as previously described [49, 50]. For data analysis, signal intensity values were filtered according to sufficient (>20 raw expression values) and significant differences in expression by GeneSpring software (analyzed by unpaired t-test - Benjamini-Hochberg correction, p-value cut-off: 0.05). Differentially expressed genes were further analyzed by gene set enrichment analysis (GSEA) and were normalized in R using Robust Multi-Array Average Normalization Approach (http://www.broadinstitute.org/gsea/msigdb/index.jsp).

### Ectopic overexpression of ABCB1 by plasmid DNA transfection

DMS114 cells were transfected with peYFP-ABCB1 plasmid DNA using XFect Transfection Reagent (Clontech, Mountain View, USA) according to the manufacturer's recommendations. peYFP-C1 served as a negative control vector. ABCB1 overexpression was monitored after 72 hours by qPCR.

### Calcein AM assay

Cells were harvested after trypsinization by centrifugation at 500x*g* and resuspended in serum-free medium. Calcein AM (0.25μM) was added to 3×10^5^ cells in 4mL medium in the presence or absence of 10μM verapamil, elacridar or nintedanib and samples incubated for 15 minutes at room temperature. Reactions were stopped by centrifugation and resuspension in ice-cold PBS. Fluorescence of intracellular Calcein was measured using a BD FACS-Calibur flow cytometer. The well-known ABCB1 modulators elacridar and verapamil were used as controls.

### ABCB1 ATPase activity

Crude cell membranes purified from Spodoptera frugiperda (Sf9) cells expressing ABCB1 were prepared as described earlier [18]. Vanadate-sensitive ATPase activity was measured by following the release of inorganic phosphate using a colorimetric reaction.

### Rhodamine (Rh123) accumulation assay

Intracellular accumulation of the ABCB1 substrate Rh123 was determined in DMS114, DMS114/NIN, KB-3-1 and KBC-1 as previously described [19]. Briefly, 5×10^5^ cells were resuspended in serum-free RPMI 1640/HEPES medium containing 3-(N-morpholino) propanesulfonic acid MOPS (Sigma) and pretreated with indicated concentrations of elacridar or nintedanib for 30 minutes at 37°C. After 1h incubation with Rh123 at room temperature, intracellular Rh123 fluorescence was measured by flow cytometry (FACSCalibur). The ABCB1 modulator elacridar was used as control.

### HPLC-MS

5×10^5^ cells were seeded in 6-well plates and incubated overnight. After 15 minutes preincubation with the selective ABCB1 inhibitor elacridar (10μM), cells were incubated with 1μM nintedanib for 10 or 60 minutes at 37°C. Transport reaction was stopped by addition of ice-cold PBS. Cells were pelleted, washed with ice-cold PBS three times and precipitated with 50μl acetonitrile (Sigma). After centrifugation at 8000xg for 5 minutes, supernatant was transferred into fresh tubes. Quantitation measurements were run on an AB Sciex 3200QTrap hybrid tandem mass spectrometer coupled to the Perkin Elmer S200 HPLC system consisting of binary pumps, autosampler and column oven. A Thermo BetaBasic C8 (50 × 3 mm, 3μm particle size) column was used for separation. Gradient elution was applied by using 0.1% formic acid in water and 0.1% formic acid in acetonitrile.

### siRNA knockdown of PKC

5×10^5^ cells were transfected with Lipofectamine 2000 (Invitrogen, Carlsbad, USA) using 50nM of PKC siRNA (Santa Cruz Biotechnology) or an equimolar concentration of scrambled siRNA (Dharmacon, Lafayette, USA) according to the manufacturer's recommendations. Downregulation of PKC expression was monitored at the protein level by Western blot 48 and 72 hours post transfection.

### Statistical analysis

If not stated otherwise, data are expressed as mean ± SD. Results were analyzed using GraphPad Prism software. Statistical analyses were performed using t-test or two-way analysis of variance (ANOVA). To examine differences between drug treatment responses, Bonferroni post-tests were conducted. P values below 0.05 were considered as statistically significant and marked with stars: * p<0.05; ** p<0.01; *** p<0.001.

## SUPPLEMENTARY MATERIALS FIGURES


