# Supplementary material for: Acquired nintedanib resistance in FGFR1-driven small cell lung cancer: role of endothelin-A receptor-activated ABCB1 expression
Source: Oncotarget. 2016 Jun 29;7(31):50161–79. doi: 10.18632/oncotarget.10324 (PMC5226575; doi:10.18632/oncotarget.10324)
Supplement: Supplementary file 1 [file oncotarget-07-50161-s001.pdf]

Acquired nintedanib resistance in FGFR1-driven small cell lung cancer: role of endothelin-A receptor-activated ABCB1 expression

SUPPLEMENTARY FIGURES

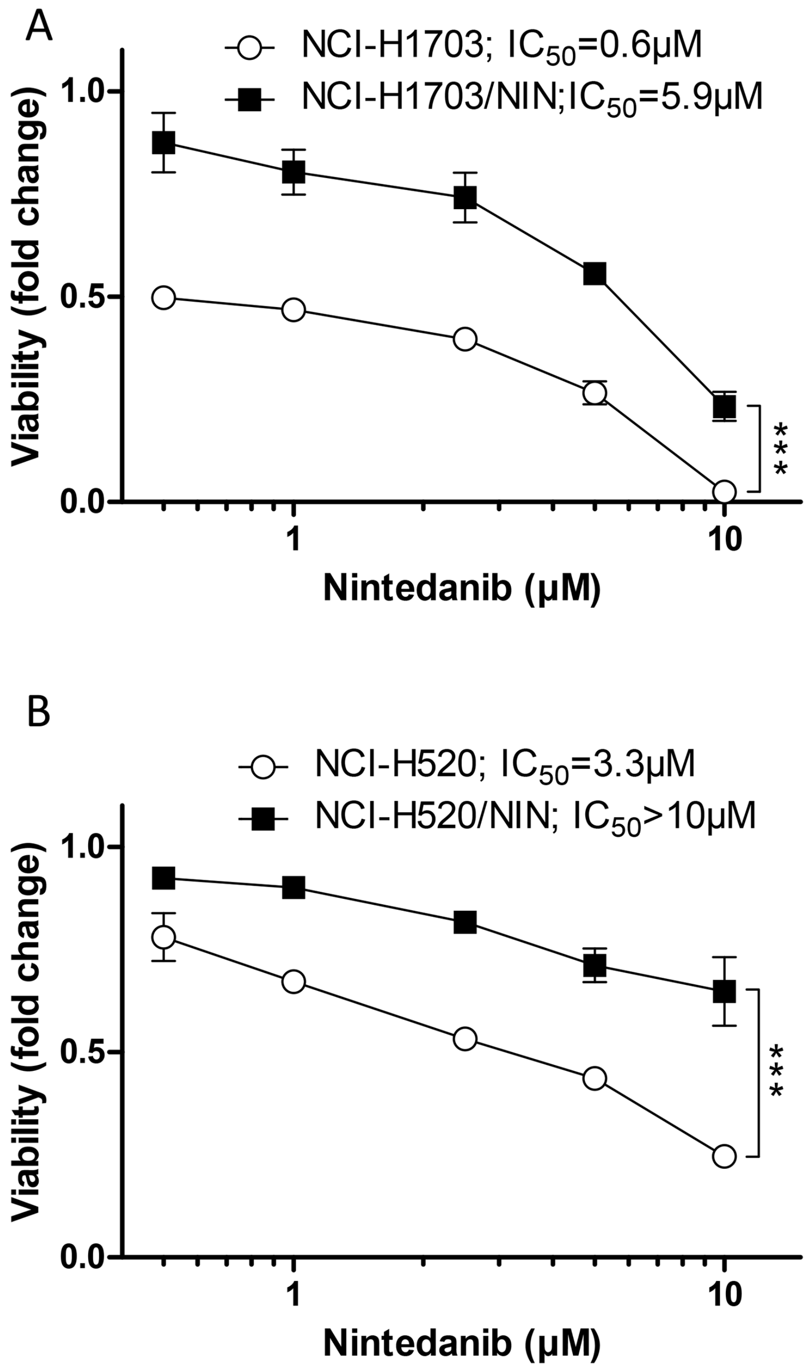

Supplementary Figure S1: Generation of two FGFR1-driven NSCLC cell lines with acquired nintedanib resistance. Viability of NCI-H1703 **A.** and NCI-H520 cells **B.** and their respective nintedanib-selected sublines was analyzed by MTT assay after 72 hours exposure to indicated concentrations of nintedanib. *** $p<0.001$, 2-way ANOVA, Bonferroni post-test.

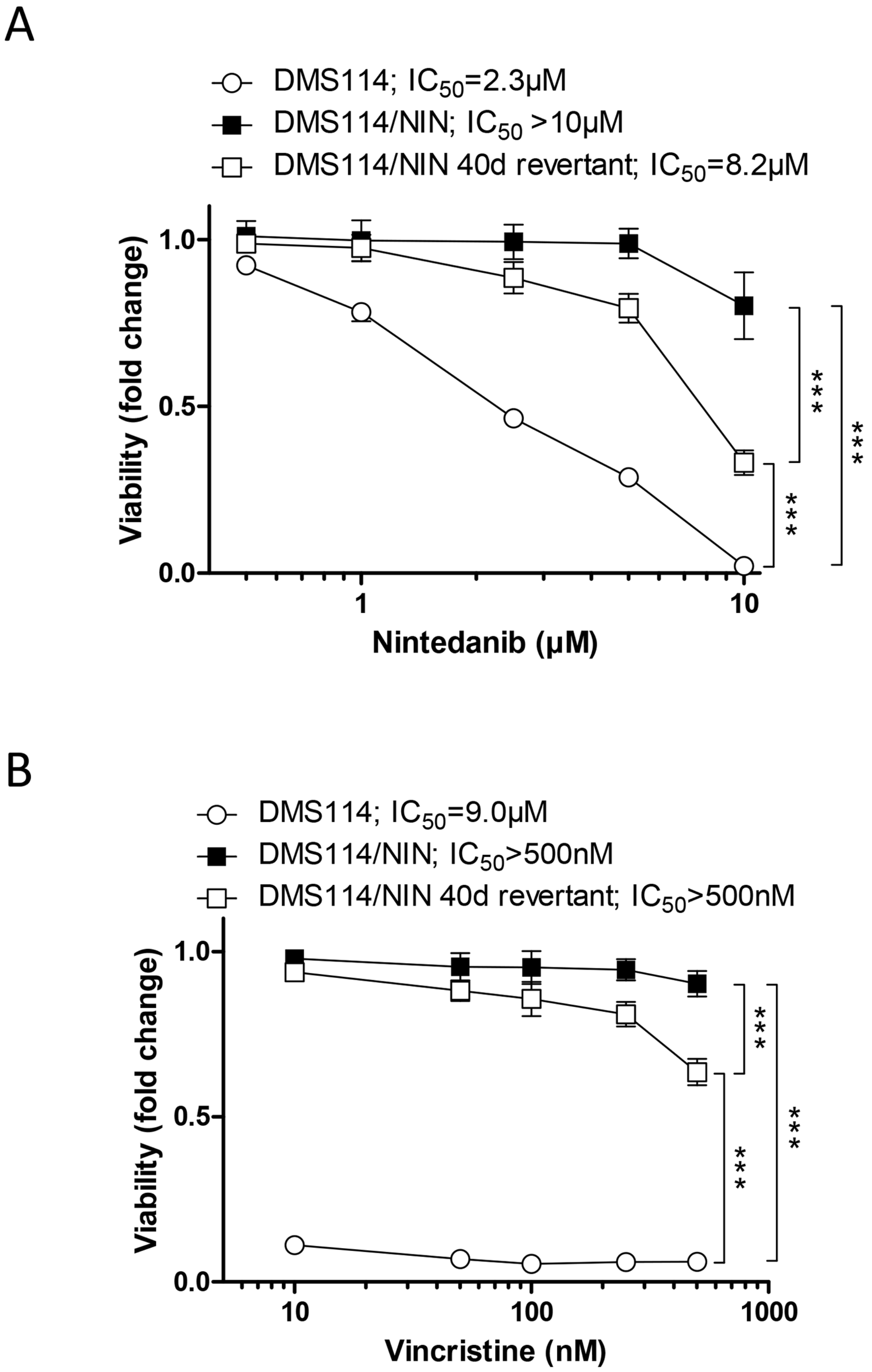

Supplementary Figure S2: Removal of nintedanib selection pressure resensitizes DMS114/NIN cells towards nintedanib and vincristine. Viability of DMS114, DMS114/NIN cells and their revertant subline, cultured for 40 days without nintedanib selection pressure, was analyzed by MTT assay after 72 hours exposure to indicated concentrations of nintedanib **A.** and vincristine **B.** *** $p<0.001$, 2-way ANOVA, Bonferroni post-test.

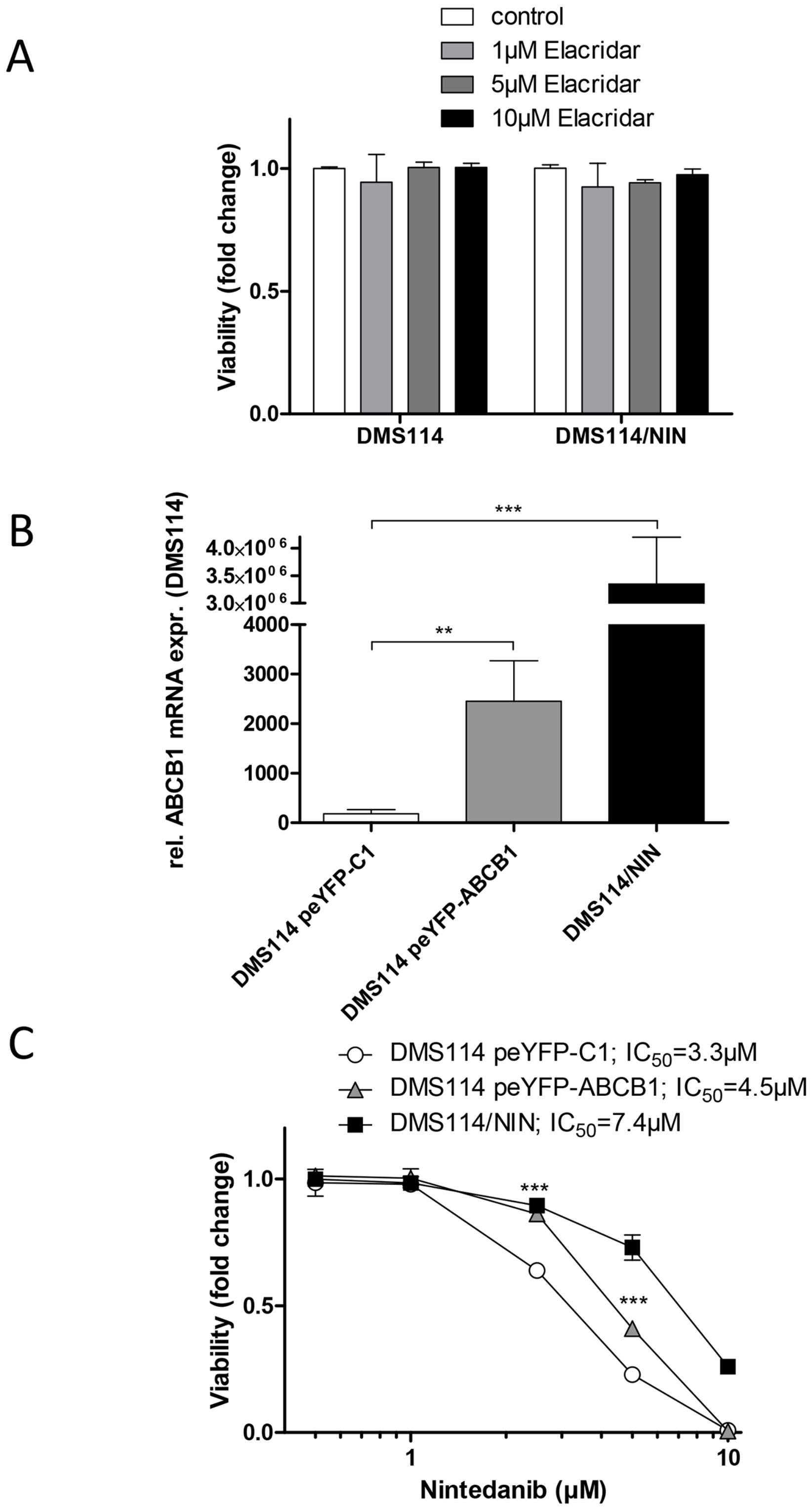

Supplementary Figure S3: DMS114 and DMS114/NIN cells are both insensitive towards elacridar. A. Viability of DMS114 and DMS114/NIN cells was analyzed by MTT assay after 72 hours exposure to indicated concentrations of the ABCB1 modulator elacridar. **B.** *ABCB1* mRNA expression was analyzed by quantitative RT-PCR and data are given normalized to *ACTB* mRNA expression. ** $p < 0.01$, *** $p<0.001$, unpaired t-test. **C.** Viability of DMS114/NIN and DMS114 cells transfected with expression vectors encoding either YFP or a YFP-ABCB1 fusion gene product was analyzed by MTT assay after 72 hours exposure to the indicated concentrations of nintedanib. *** $p < 0.001$, 2-way ANOVA, Bonferroni post-test.

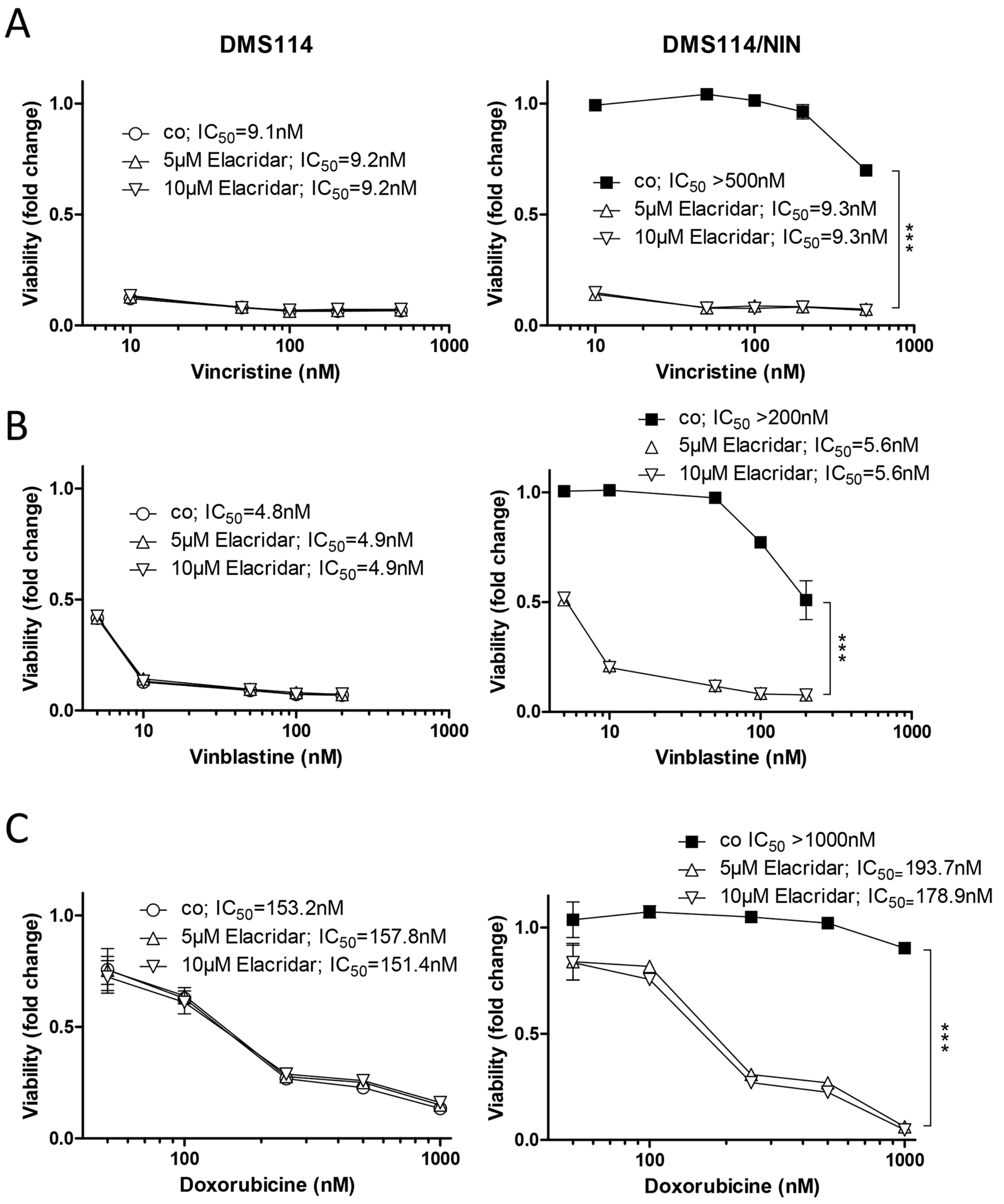

Supplementary Figure S4: Selection of DMS114 against nintedanib causes ABCB1-mediated multidrug resistance. Viability of DMS114 and DMS114/NIN cells was analyzed by MTT assay after 72 hours exposure to indicated concentrations of vincristine **A.** vinblastine **B.** and doxorubicin **C.** in the presence or absence of indicated concentrations of the ABCB1 modulator elacridar. *** $p<0.001$, 2-way ANOVA, Bonferroni post-test.

A

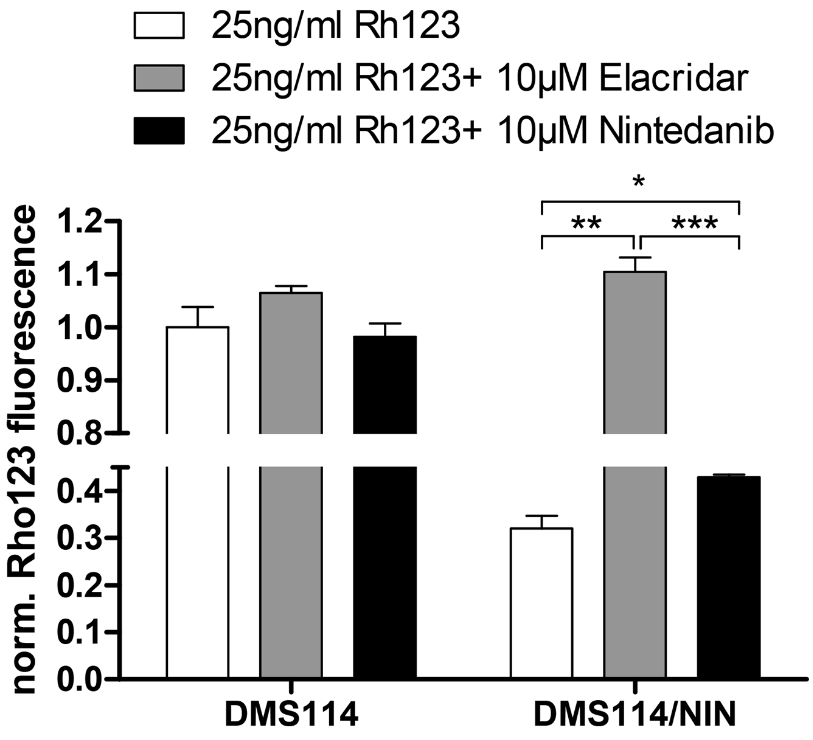

B

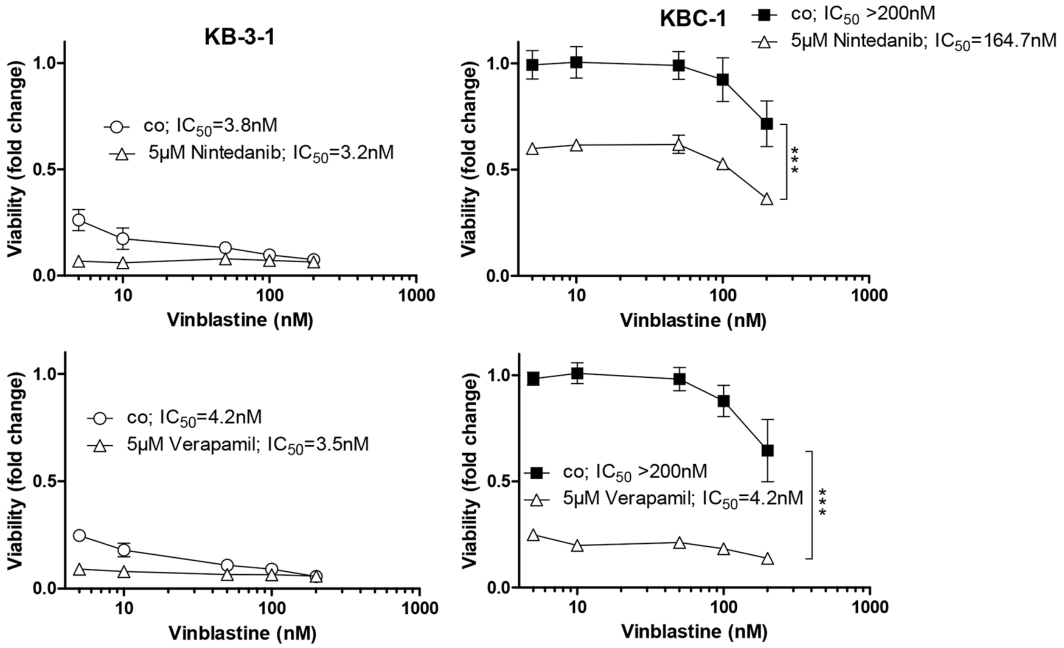

Supplementary Figure S5: Nintedanib increases intracellular accumulation of the ABCB1 substrate Rhodamin123 (Rh123) in DMS114/NIN and KBC-1 cells. A. To analyze the impact of nintedanib on intracellular accumulation of the fluorescent ABCB1 substrate Rh123, DMS114 and DMS114/NIN cells were incubated with 0.25ng/ml Rh123 for 60min in the presence or absence of 10μM nintedanib. Intracellular Rh123 fluorescence was measured by FACS and analyzed by FlowJo software. The ABCB1 modulator elacridar served as positive control. * $p < 0.05$, ** $p < 0.01$, *** $p < 0.001$, unpaired t-test. **B.** Viability of KB-3-1 and KBC-1 was analyzed by MTT assay after 72 hours exposure to vinblastine in the presence or absence of 5μM nintedanib. Verapamil (*lower panels*) was used as positive control for ABCB1 modulation. *** $p<0.001$, 2-way ANOVA, Bonferroni post-test.

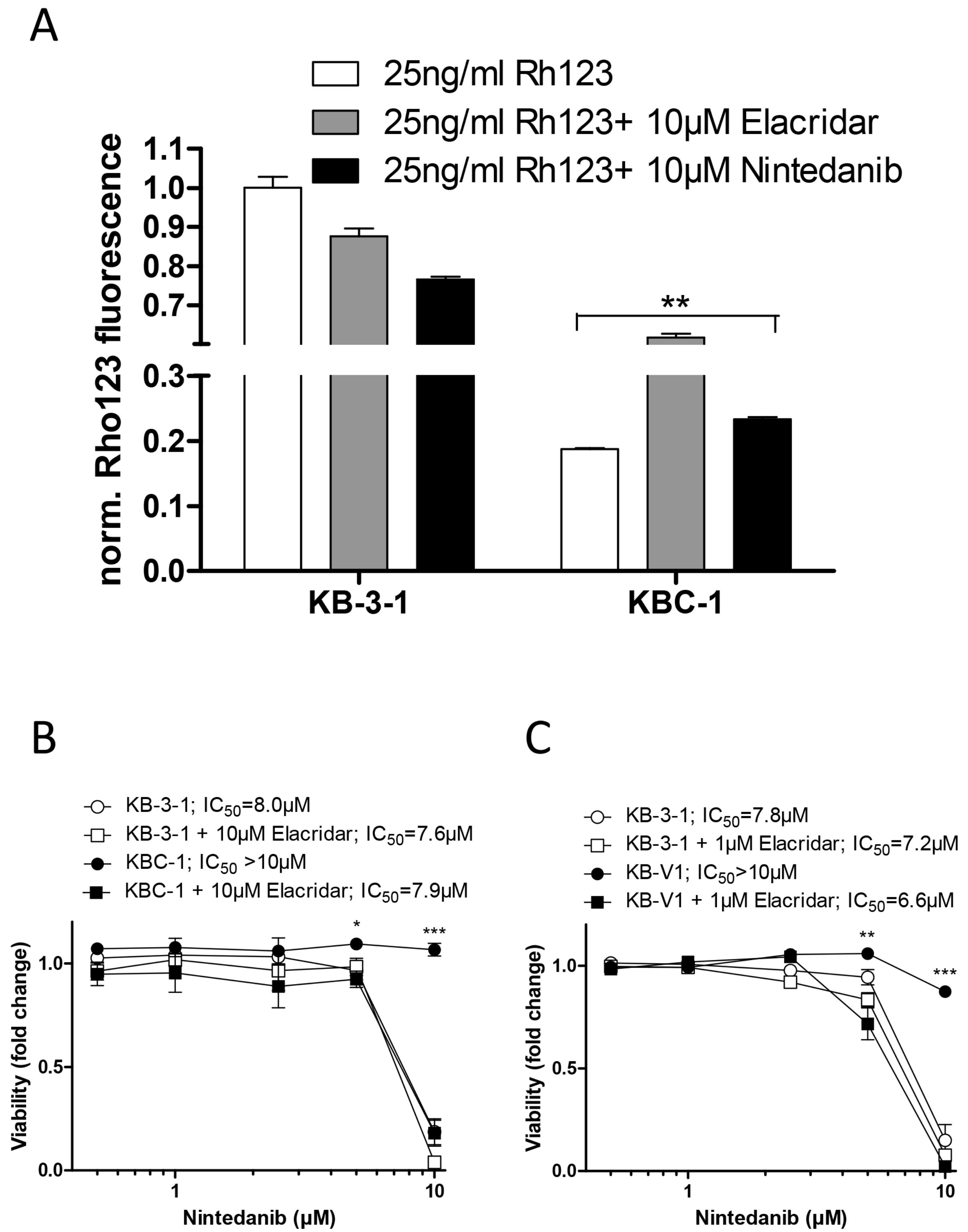

Supplementary Figure S6: Inhibition of ABCB1 resensitizes KBC-1 cells to nintedanib. A. To analyze the impact of nintedanib on intracellular accumulation of the fluorescent ABCB1 substrate Rh123, KB-3-1 and KBC-1 cells were incubated with 0.25ng/ml Rh123 for 60 min in the presence or absence of 10µM nintedanib. Intracellular Rh123 fluorescence was measured by FACS and analyzed by FlowJo software. The ABCB1 modulator elacridar served as positive control. ** $p < 0.01$, unpaired t-test. Viability of KB-3-1 and KBC-1 **B.** as well as KB-V1 **C.** cells was analyzed by MTT assay after 72 hours exposure to indicated concentrations of nintedanib in the presence or absence of elacridar. * $p < 0.05$, ** $p < 0.01$, *** $p < 0.001$, unpaired t-test.

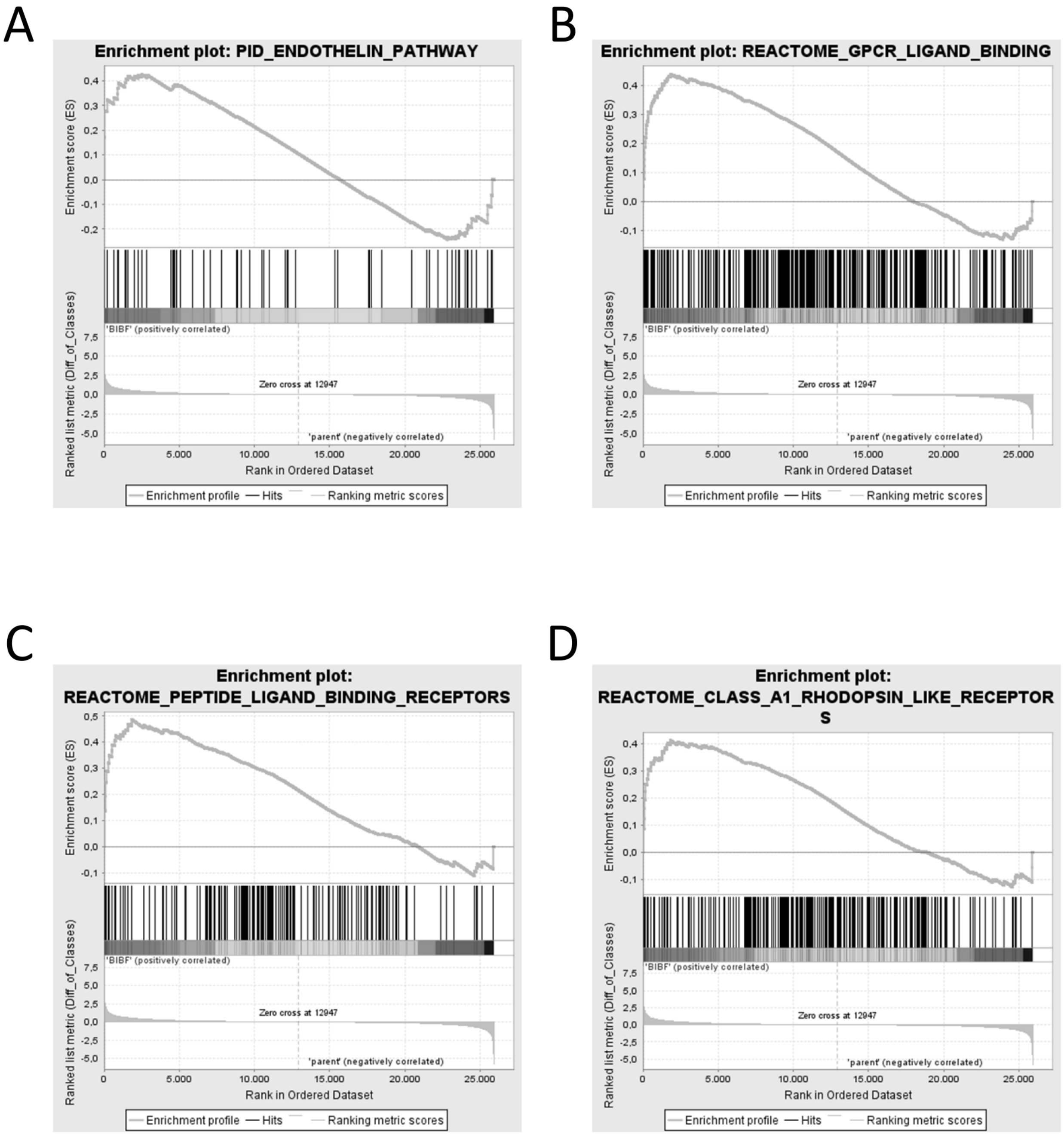

Supplementary Figure S7: Gene set enrichment analysis (GSEA) reveals upregulation of endothelin signaling members in DMS114/NIN cells. GSEA analysis of whole-genome gene expression data showed enrichment of the PID endothelin-pathway **A.** reactome GPCR ligand binding **B.** peptide ligand binding receptors **C.** and class A1 rhodopsin-like receptors **D.** datasets, all containing both endothelin-1 and endothelin-A receptor genes.

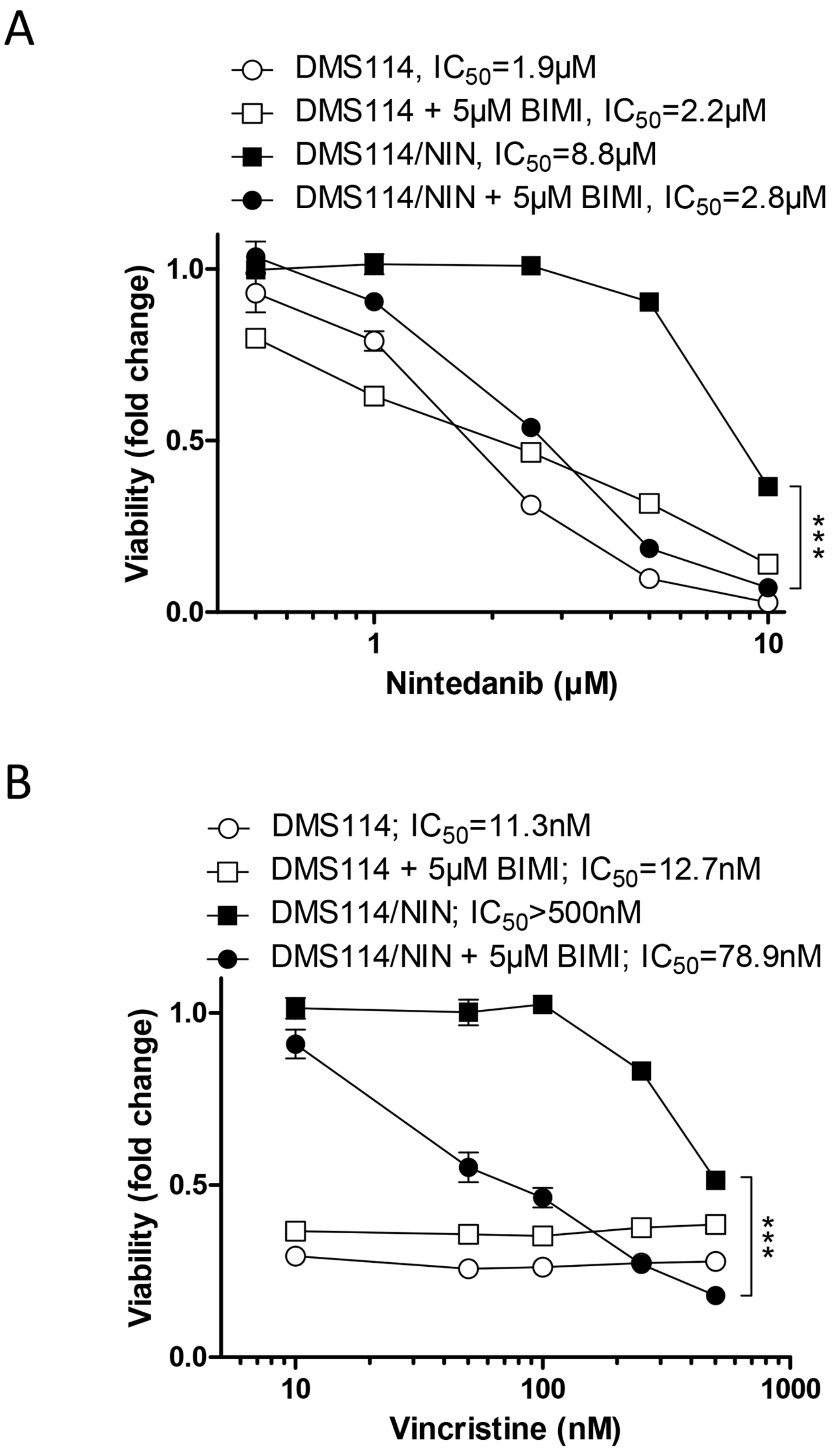

Supplementary Figure S8: Inhibition of PKC resensitizes DMS114 cells towards nintedanib and vincristine. Viability of DMS114 and DMS114/NIN cells, 72 hours pretreated with 5μM of the PKC inhibitor BIMI, was analyzed by MTT assay after 72 hours exposure to indicated concentrations of nintedanib **A.** and vincristine **B.**
